# ERLD-HC: Entropy-Regularized Latent Diffusion for Harmony-Constrained Symbolic Music Generation

**DOI:** 10.3390/e27090901

**Published:** 2025-08-25

**Authors:** Yang Li

**Affiliations:** School of Science, China University of Petroleum (Beijing), Beijing 102249, China; ly.cup.research@gmail.com

**Keywords:** symbolic music generation, entropy-regularized CRF, latent diffusion, harmonic control, variational autoencoder

## Abstract

Recently, music generation models based on deep learning have made remarkable progress in the field of symbolic music generation. However, the existing methods often have problems of violating musical rules, especially since the control of harmonic structure is relatively weak. To address these limitations, this paper proposes a novel framework, the Entropy-Regularized Latent Diffusion for Harmony-Constrained (ERLD-HC), which combines a variational autoencoder (VAE) and latent diffusion models with an entropy-regularized conditional random field (CRF). Our model first encodes symbolic music into latent representations through VAE, and then introduces the entropy-based CRF module into the cross-attention layer of UNet during the diffusion process, achieving harmonic conditioning. The proposed model balances two key limitations in symbolic music generation: the lack of theoretical correctness of pure algorithm-driven methods and the lack of flexibility of rule-based methods. In particular, the CRF module learns classic harmony rules through learnable feature functions, significantly improving the harmony quality of the generated Musical Instrument Digital Interface (MIDI). Experiments on the Lakh MIDI dataset show that compared with the baseline VAE+Diffusion, the violation rates of harmony rules of the ERLD-HC model under self-generated and controlled inputs have decreased by 2.35% and 1.4% respectively. Meanwhile, the MIDI generated by the model maintains a high degree of melodic naturalness. Importantly, the harmonic guidance in ERLD-HC is derived from an internal CRF inference module, which enforces consistency with music-theoretic priors. While this does not yet provide direct external chord conditioning, it introduces a form of learned harmonic controllability that balances flexibility and theoretical rigor.

## 1. Introduction

In recent years, music generation models based on artificial intelligence have made remarkable progress. Deep learning models [1,2] have demonstrated outstanding capabilities in learning and generating complex music. Neural network models such as MusicVAE [3] and music transformer models [4] have proven their effectiveness in symbolic music generation by establishing models of music structures and generating coherent melody sequences. Transformer-based frameworks, for instance, Hybrid Learning Transformer [5], which integrates music theory modules, and Structured Music Transformer [6], which fuses style clustering for conditional generation. Meanwhile methods like Musika! [7] and Compound Word Transformer [8] continue to expand the boundaries of symbolic generation. However, despite these advancements, existing approaches still face notable limitations.

Large language models (LLMs) applied to music, such as MusicBERT [9] and ChatMusician [10], have demonstrated potential in tasks like melody generation and complex musical pattern modeling through pretraining on symbolic music data. However, these models often lack explicit modeling of musical structure during the generation process, resulting in insufficient consistency in harmony, rhythm, and style. Although diffusion-based models for symbolic music generation [11,12] can generate high-quality musical sequences, they also typically lack clear mechanisms to embed music theory rules. More recent works further extend this direction. Mustango [13] incorporates text-prompt controllability into symbolic diffusion, enabling semantic conditioning but not explicitly modeling harmonic rules. Hierarchical Cascaded Diffusion [14] improves long-range coherence through multi-scale scheduling, but its control remains limited to structural levels. In the audio domain, MusicLDM [15] demonstrates text-conditioned diffusion for waveform music generation, but it cannot be directly applied to symbolic tasks. These methods highlight the growing interest in controllable diffusion for music, yet they do not provide a principled mechanism for enforcing harmonic consistency in symbolic generation.

Rule-guided diffusion approaches [16] introduce musical rules to guide generation, but they often suffer from limited flexibility and reduced diversity due to strict human design constraints. The contradiction between theoretical rigor and creative diversity in music algorithms is obvious in the existing hybrid models. The hierarchical RNN proposed by Zixun et al. [17] achieved end-to-end symbolic melody generation with coherent long-term structure via a hierarchical strategy. Although this model performs well in terms of global structure control, it still has the problem of rhythmic rigidity at the local note level, highlighting what can be described as an over-constraint problem in the rule-based architecture of music generation. Conversely, the Structure-Informed Transformer [18] bypassed explicit symbolic rules by embedding music-theoretical structures as relative positional encodings. This can enhance the rhythmic resolution, but due to its insufficient simulation of vertical sound constraints, it usually violates the voice part rules, such as parallel octaves. In contrast, DeepBach [19], a neuro-symbolic model that integrated strict counterpoint rules with neural networks, achieves highly stylistic and accurate chorale generation, but strictly adheres to counterpoint rules, making it stylistically rigid and limited to Bach chorales. These strategies embody the approximation dilemma in music generation, especially harmony generation, where purely statistical models often compromise harmonic coherence, and symbolic rule systems, though accurate in theory, restrict creative freedom through overly rigid constraints.

To overcome the above challenges, we propose a novel symbolic music generation framework, Entropy-Regularized Latent Diffusion for Harmony-Constrained (ERLD-HC), that integrates a variational autoencoder (VAE)-diffusion hybrid architecture with entropy-regularized harmonic constraints. Specifically, we enhance the cross-attention mechanism within the diffusion process by injecting chord-aware representations learned via a Conditional Random Field (CRF) [20], guided by entropy-based regularization. This design enables the model to incorporate music-theoretic priors while maintaining flexibility and diversity in melodic generation. Unlike traditional rule-based systems or purely data-driven models, our approach introduces a learnable harmonic prior that improves the coherence and consistency of generated music. Our main contributions are as follows:

1. We propose a hierarchical generation architecture, ERLD-HC, which combines a VAE-based latent space representation with a denoising diffusion model, leveraging the strengths of both generative paradigms for symbolic music generation.
2. We introduce an entropy-guided CRF module into the cross-attention of the diffusion model, enabling the incorporation of interpretable harmonic priors and the regulation of chord progression in a differentiable manner. This design enhances the harmonic coherence of generated sequences through internal inference, without requiring external chord labels or explicit chord conditioning.

The significance of our method lies in its ability to reconcile two traditionally conflicting goals in music generation: the creative flexibility provided by neural networks and the strict rules of music theory. By embedding entropy-regularized, learnable music-theoretic constraints into the cross-attention mechanism of the diffusion process, we establish a new deep learning framework that respects theoretical rigor while preserving expressive capacity.

## 2. Data Preprocessing

This paper adopts Musical Instrument Digital Interface (MIDI) files for the representation of music sequences. MIDI is a symbolic format that stores musical control and structural information rather than actual audio signals, making it suitable for digital composition and algorithmic analysis. A MIDI file typically consists of two main components: the header block, which includes metadata such as file type, number of tracks, and temporal resolution; and track blocks, which store a sequence of MIDI events. These events include note events (Note On/Off) that indicate the start and end of a note, with associated pitch and timing information; control changes that modify parameters such as volume or modulation; program change that switches instrument timbres; and meta-events that encode additional information like time signatures, lyrics, and key.

**Figure 1:**
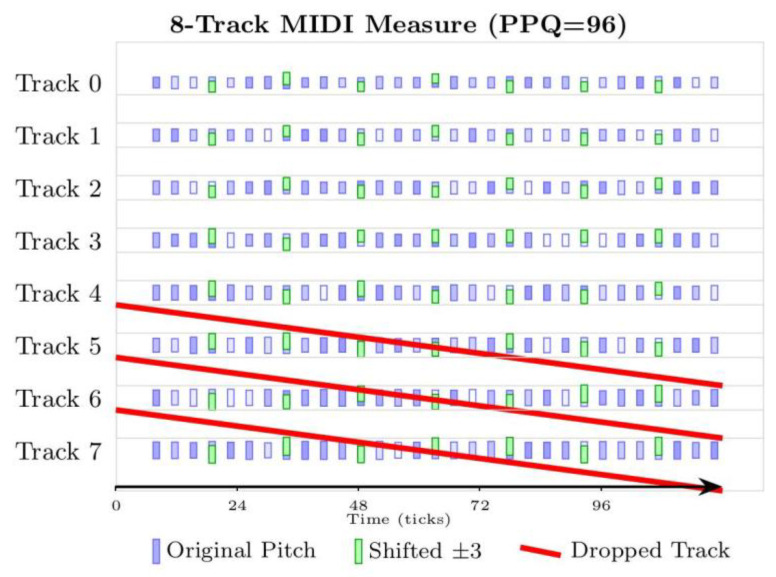
MIDI file data processing. Time offset is shown along the horizontal axis, while the vertical axis indicates the presence of eight distinct tracks. Within each track, the height of a rectangle indicates the pitch of the note event. The purple rectangle represents the original pitch, the depth of the color indicates the intensity, the green rectangle represents the pitch offset, and the red rectangle represents the randomly dropped track.

To facilitate the input of symbolic music into the model, this paper refers to MusicVAE to convert the note-level information, such as duration, pitch, start time, end time, dynamics, etc., in the MIDI file into a standardized data format. Specifically, the pitch in each MIDI file is encoded as 0-127. The intensity of the MIDI file is quantified into eight intervals, each interval representing a specific volume range. Each volume change event sets a segment, in which all subsequent note-on events will use this set volume level until another volume change event occurs. The advancement of time is represented by 96 time offset events, and each event advances the current time by one quantified time step. Twenty-four time steps represent the length of a quarter note. In addition to the 128 different instrument sounds defined by the MIDI standard, a number channel for drums has also been added, with a total of 129 program selection events. The MIDI program number is set at the beginning of each track. The model we proposed models musical segments that contain up to eight tracks, with each track consisting of a single instrument, with a globally unified clock resolution of 96 pulses per quarter note (PPQ). To address the unbalanced style distribution in the dataset, this paper adopts a data enhancement strategy. The pitch of notes is randomly offset by up to ±3 semitones. To maintain tonal consistency, offsets are applied such that resulting pitches remain within the current key scale: if a pitch after randomization falls outside the scale, it is mapped to the nearest scale tone. This ensures harmonic coherence while introducing small variations for expressive diversity, and the global dynamics are scaled by ±15% to simulate the dynamic changes in performance. To improve model robustness, we randomly discard one to two non-primary melody tracks during augmentation. Non-primary melodies are defined as accompaniment or auxiliary parts that typically exhibit lower pitch variance, sparser note density, and less dynamic variation. Using Music21, each track is analyzed for these characteristics to identify the primary melody; remaining tracks are treated as non-primary. This selective removal maintains the integrity of the main melody, as shown in Figure 1.

## 3. Methods

### 3.1. Model Network Structure

**Figure 2:**
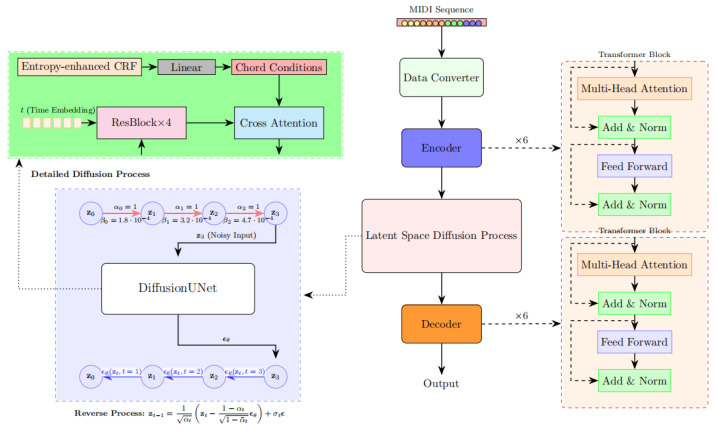
The structure of the ERLD-HC model.

The overall architecture of the proposed ERLD-HC model is illustrated in Figure 2. Our model adopts a transformer-based VAE backbone. The encoder consists of six transformer layers [21], with each comprising an eight-head self-attention mechanism (with a hidden dimension of 512) and a feedforward network (FFN) with an intermediate dimension of 2048. The encoder outputs the mean and variance of the latent variables, from which a 256-dimensional latent code z is sampled using the reparameterization trick.

A diffusion process is then applied in the latent space. In the forward process, Gaussian noise is added to the latent code z using a cosine noise schedule. In the reverse process, a UNet-based noise predictor [22], composed of four residual blocks [23] and attention layers, gradually denoises the latent representation.

To ensure the generated output conforms to symbolic music structures and emphasizes harmonic correctness rather than audio fidelity, we retain the VAE decoder to preserve constraint consistency throughout the generation. Decoder takes the latent representation produced by the diffusion module as input and generates pitch, duration, velocity, etc. Unlike autoregressive decoders, our design does not apply causal masking, allowing the model to leverage global context during decoding and enabling efficient generation of complete musical segments.

Importantly, we introduce an entropy-regularized CRF mechanism into the cross-attention layers of the diffusion network, enabling the model to integrate harmony-aware constraints in a learnable and differentiable manner during generation.

### 3.2. VAE-Encoder

Each MIDI file is first split into bars based on time-change boundaries. Typically, 4 bars are included per training segment, similar in spirit to MusicVAE’s segmentation approach. These bar-level sequences are then converted into TFRecord format. Sequences that do not meet predefined conditions (e.g., invalid pitch, too few notes) are filtered out based on parameters. A data converter is used to convert the TFRecord sequences into tensors, which are subsequently fed into the VAE encoder. As shown in Equation (1), $E_{midi}\left(x\right)$ represents a 128-dimensional feature vector extracted from the original MIDI sequence, containing quantized pitch, timing, and dynamic information.

(1) $$z=Encoder(TFRecord\left[E_{midi}\left(x\right)\right])$$

### 3.3. Latent Diffusion

Encoder output $z_{0}\in R^{128}$ is directly used as the initial latent variable in the diffusion process. The diffusion is divided into the forward noise-adding process and the reverse denoising process. In the forward noise-adding process, Gaussian noise [24] is gradually added to obtain $z_{1},z_{2}\ldots z_{t}$ from $z_{0}$, as defined in Equation (2):

(2) $$z_{t}=\sqrt{\alpha_{t}}z_{t-1}+\sqrt{1-\alpha_{t}}\epsilon ,\epsilon \sim Ν\left(0,I\right)$$

Here, $\alpha_{t}$ denotes the improved cosine-based noise schedule. The noise scheduling coefficient $\alpha_{t}$ is used to control the intensity of noise addition. In the initial stage, the original signal is largely preserved, and complete noise reduction is achieved in the final stage. The cosine function changes gently within the interval $\left[0,\pi /2\right]$, avoiding sudden changes in linear scheduling at both ends. It is suitable for music signals because the harmonic structure needs to be smoothly disrupted. Meanwhile, stable scheduling is conducive to the effective transmission of CRF constraints. The scheduling function is defined as Equation (3):

(3) $$\alpha_{t}={cos}^{2}\left(\frac{t/T+s}{1+s}\cdot \frac{\pi }{2}\right)$$

t denotes the current timestep, and T is the total number of diffusion steps. The offset s=0.008, the reverse denoising process uses a trained UNet to predict the noise $\varepsilon_{\theta }\left(z_{t},t\right)$, gradually restoring $z_{0}$ from $z_{t}$, as shown in Equation (4):

(4) $$z_{t-1}=\frac{1}{\sqrt{\alpha_{t}}}\left(z_{t}-\frac{1-\alpha_{t}}{\sqrt{1-\bar{\alpha_{t}}}}\epsilon_{\theta }\right)+\sigma_{t}\epsilon$$

$\epsilon_{\theta }$ is the noise predicted by UNet, see blue arrow in Figure 2. The core modules of the UNet architecture include four resblocks and cross-attention. The chord sequence output by entropy-enhanced CRF is mapped to a 128-dimensional condition vector through linear projection. In each denoising step of the diffusion process, the chord features generated by CRF are introduced through cross-attention, thereby constraining the generation results at the latent space level. It enables dynamic alignment between latent note sequences and chord conditions. Compared with simple concatenation, cross-attention can retain the temporal relationship between the two sequences and avoid information confusion. At each layer of UNet, the diffusion intermediate noise $z_{t}$ is used as the Query, and the chord condition vector *c* is used as the Key and Value; the conditioning vector c represents the harmonic conditioning embedding. Specifically, we first apply the entropy-regularized CRF module to infer chord labels from the latent pitch-class sequence of each bar. Each chord label is then mapped into a dense embedding through a learnable embedding matrix E. The resulting condition vector $c\in R^{d}$ represents a chord-aware harmonic embedding. During training, the parameters of the embedding matrix E are optimized jointly with the diffusion model, so that the chord embeddings become increasingly aligned with the generation objective. At each denoising step, given the latent feature representation $z_{t}$, we compute the query, key, and value as follows:

(5) $$Q=W_{q}\cdot z_{t},\;K=W_{k}\cdot c,\;V=W_{v}\cdot c$$

$W_{q},W_{k},W_{v}\in R^{d\times d}$ is the learnable projection matrix. Cross-attention integrates the attended output into the residual connection and then pass through LayerNorm (LN); the process is shown as Equation (6):

(6) $$z_{t+1}=LN\left(z_{t}+W_{o}\cdot softmax\left(\frac{QK^{T}}{\sqrt{d}}\right)V\right)$$

$W_{o}\in R^{d\times d}$ is the output projection matrix. Cross-attention dynamically highlights relevant chord features at each denoising step. This weighted fusion ensures harmonic consistency without disrupting the inherent noise-prediction dynamics of the diffusion model. LN and residual connections stabilize training, as the attention output is added to the original feature map before normalization. Crucially, this approach outperforms concatenation by preserving long-range dependencies: chords influence note generation adaptively, even when sequences are non-aligned. We use a 128-dimensional projection to preserve harmonic context while maintaining computational efficiency, avoiding unnecessary overparameterization. In order to enable the model to utilize external information such as harmony on features at different levels, cross-attention layers are introduced within each intermediate UNet block.

### 3.4. Chord Inference Based on Entropy-Regularized CRF

In the music generation model, chord inference critically determines the structural coherence and theoretical rationality of the synthesized MIDI signals. Traditional probabilistic models such as Hidden Markov models [25] are limited by the assumption of local transitions, making them inadequate for modeling long-range harmonic dependencies and tonal structures. To overcome this limitation, we adopt a CRF framework enhanced by entropy-based feature functions [26], which encode both data-driven and theory-aware properties into a unified probabilistic model.

CRF is a discriminative model used for sequence labeling tasks [27], which calculates the conditional probability distribution over label sequences given an observation sequence. Unlike traditional models, CRF allows the incorporation of complex, dependent feature functions. We reformulate these functions using entropy measures to capture harmonic consistency, tonal stability, and melodic smoothness in a statistically grounded manner.

Unlike classifier guidance or classifier-free guidance, our framework does not expose an external chord-conditioning interface to the user. Instead, harmonic controllability is achieved implicitly through the CRF module, which infers chord labels from the latent pitch distribution and integrates them into the diffusion process via cross-attention. Thus, “controllability” in our work should be interpreted as internal harmonic regulation guided by CRF inference, rather than explicit user control with external chord labels.

Given an input sequence of unit-normalized pitch class vectors $\left\{y_{1},y_{2},\ldots y_{T}\right\}$, where each vectors $y_{t}$ is derived from weighted pitch occurrences in a bar, the conditional probability of a chord label sequence $h=\left\{h_{1},h_{2},\ldots ,h_{T}\right\}$ is defined as:

(7) $$p\left(h\left|y\right.\right)=\frac{1}{Z\left(y\right)}exp\left(\sum_{t=1}^{T}\sum_{k}\lambda_{k}f_{k}\left(h_{t-1},h_{t}{,y}_{t}\right)\right)$$

Here, $f_{k}$ denotes the *k*-th entropy-based feature function, and $\lambda_{k}$ is its learnable weight. $Z\left(y\right)$ is the partition function that normalizes the distribution. We propose the following four types of entropy-based feature functions.

#### 3.4.1. Pitch Class Entropy(PCE)

To evaluate how concentrated a pitch vector $y_{t}$ is around certain chord tones, we define a pitch entropy function as Equation (8):

(8) $$f_{PCE}\left(h_{t},y_{t}\right)=-\sum_{i=1}^{12}p_{i}logp_{i}$$

(9) $$p_{i}=\frac{{count}_{i}+1}{\sum_{j=1}^{12}\left({count}_{j}+1\right)}$$

Here, ${count}_{i}$ denotes the number of occurrences of pitch class i within a bar, $i\in \left\{1,\ldots ,12\right\}$. The additive constant ensures Laplace smoothing, avoiding zero probabilities and stabilizing the entropy estimation. A lower entropy indicates tonal concentration, which is more likely to match standard chord structures.

We adopt the 12-tone equal temperament (12-TET) representation, where pitch classes are indexed from 1 to 12:

1 = *C*, 2 = *C*#, 3 = *D*, 4 = *D*#, 5 = *E*, 6 = *F*, 7 = *F*#, 8 = *G*, 9 = *G*#, 10 = *A*, 11 = *A*#, 12 = *B* (10)

This convention is consistently used in subsequent entropy functions.

#### 3.4.2. Chord Transition Entropy (CTE)

Instead of using binary chord change indicators, we introduce a statistical prior over chord transitions. Let ∁ denote the set candidate chords, here restricted to the 24 major/minor triads commonly used in tonal harmony. For two consecutive time steps $h_{t-1},h_{t}\in ∁$, we define the smoothed empirical transition probability as:

(11) $$P\left(h_{t}|h_{t-1}\right)=\frac{N\left(h_{t-1},h_{t}\right)+\alpha }{\sum_{h^{\prime }\in ∁}\left(N\left(h_{t-1},h^{\prime }\right)+\alpha \right)}$$

where $N\left(h_{t-1},h_{t}\right)$ denotes the number of times the transition from chord $h_{t-1}$ to chord $h_{t}$ occurs in the training corpus, and α=1 is a Laplace smoothing constant to avoid zero probabilities for unseen transitions.

The feature function is then defined as:

(12) $$f_{CTE}\left(h_{t},h_{t-1}\right)=-logP\left(h_{t}|h_{t-1}\right)$$

which penalizes rare or disharmonious transitions, thereby enforcing chord progressions that are more typical of tonal music. The entropy is computed across successive bars, capturing long-range harmonic dependencies beyond local pitch-class statistics.

#### 3.4.3. Key Matching Entropy (KME)

To assess the alignment between the current chord $h_{t}$ and the tonal context $k_{t}$, we define a key-alignment entropy in Equation (13):

(13) $$f_{KME}\left(h_{t},k_{t}\right)=-\sum_{i=1}^{12}q_{i}logq_{i}$$

where $q_{i}$ is the normalized probability that pitch class *i* both occurs in chord $h_{t}$ and belongs to the diatonic scale of the key $k_{t}$. Formally,

(14) $$q_{i}=\frac{B_{i}\left(h_{t}\right)\cdot Ι\left(i\in scale\left(k_{t}\right)\right)+\alpha }{\sum_{j=1}^{12}\left(B_{j}\left(h_{t}\right)\cdot Ι\left(j\in scale\left(k_{t}\right)\right)\right)+\alpha }$$

With $B_{i}\left(h_{t}\right)=1$ if pitch class *i* occurs in chord $h_{t}$, and 0 otherwise, $Ι\left(\cdot \right)$ the indicator function defined as:

(15) $$Ι\left(i\in scale\left(k_{t}\right)\right)=\left\{\begin{array}{c} 1,if\;pitch\;class\;i\;belongs\;to\;the\;diatonic\;scale\;of\;key\;k_{t}\\ 0,otherwise \end{array}\right.$$

Here, α=1 is a smoothing constant to avoid zero probabilities, the diatonic scale of $k_{t}$ is defined according to Western tonal theory in 12-TET representation. For example: C major key contains pitch classes at scale degrees $\left\{1,3,5,6,8,10,12\right\}$ relative to its tonic. A minor key contains pitch classes $\left\{10,12,1,3,5,6,8\right\}$ relative to its tonic.

A low-entropy value implies the chord is harmonically consistent with the current key, while non-diatonic tones increase entropy, reflecting harmonic tension.

#### 3.4.4. Tonal Entropy (TE)

To avoid erratic key modulations, we introduce a penalty on tonal inconsistency across time as follows:

(16) $$f_{TE}\left(k_{t},k_{t-1}\right)=Ι\left(k_{t}\neq k_{t-1}\right)\cdot H\left(K\right)$$

(17) $$H\left(Κ\right)=-\sum_{k}P\left(k\right)logP\left(k\right)$$

Here, $H\left(Κ\right)$ is the entropy of the key distribution over the full sequence. With *K*, the set of possible keys. The probability *P*(*k*) is estimated from corpus-level counts with Laplace smoothing:

(18) $$P\left(k\right)=\frac{N\left(k\right)+\alpha }{\sum_{K^{\prime }\in Κ}N\left(k^{\prime }+\alpha \right)}$$

Here, α=1, *N*(*k*) denotes the number of times key *k* appears in the training corpus. High entropy implies frequent modulations, which are penalized unless musically justified.

Finally, all feature weights $\lambda_{k}$ are optimized by maximizing the log-likelihood of the correct chord sequence in Equation (19):

(19) $$L=logΡ\left(h^{true}\left|y\right.\right)$$

where the partition function is computed implicitly via dynamic programming (forward–backward), through this entropy-regularized CRF, the model captures global tonal stability while allowing flexible local variations, achieving a balance between music-theoretic soundness and creative expressiveness.

#### 3.4.5. CRF Training and Integration with Diffusion

While the previous sections define the entropy-based feature functions and associated probabilities, it is important to clarify the training and integration of the CRF with the diffusion model. The CRF module is first trained independently in a pretraining stage; given the pitch-class sequence of each bar, the CRF optimizes the log-likelihood of the correct chord sequence, with the partition function Z(y) computed efficiently via the forward–backward algorithm. This ensures proper normalization over all possible chord sequences and stable probability estimates. Smoothing constants α are applied as described in Section 3.4.1, Section 3.4.2, Section 3.4.3 to handle rare or unseen events.

After pretraining, the CRF parameters are frozen during diffusion training to guarantee stable chord inference and to avoid interference from noisy diffusion gradients. The inferred chord sequences are mapped to learnable embeddings and serve as harmonic conditioning vectors for the diffusion model. During each denoising step of the latent diffusion process, these chord embeddings are used as the Key and Value in the cross-attention layers, while the intermediate latent feature $z_{t}$ serves as the Query. This allows the diffusion model to dynamically align the generated note sequences with harmonically plausible chord structures, enforcing long-range tonal consistency without constraining local creative variations.

The choice of CRF-derived features over simpler alternatives (e.g., raw pitch-class vectors or probability vectors) is motivated by their ability to explicitly encode music-theory-informed constraints such as tonal stability, chord transition probabilities, and key alignment. Simple features capture only local or instantaneous distributions, lacking the temporal and structural context necessary for harmonically coherent generation. By contrast, entropy-regularized CRF outputs provide a robust, theory-aware conditioning signal that guides the diffusion model while preserving expressive flexibility.

### 3.5. VAE-Decoder

After the denoising process of the diffusion model, the final denoised latent sequence $z_{0}\in R^{T\times D}$ encapsulates the musical structure and harmonic information conditioned by prior chord-aware modeling. The decoder is designed to transform this latent representation into structured event sequences, which are then converted into symbolic MIDI format.

We adopt a transformer-based decoder to parameterize the output distribution over musical event tokens, as shown in Equation (20):

(20) $$P\left(e_{t}\left|z_{0}\right.\right)=Decoder{\left(z_{0}\right)}_{t}$$

Here, $e_{t}$ denotes the event token at time step t, and the decoder outputs a probability distribution over the vocabulary of discrete musical events (e.g., note-on, note-off, velocity, duration). The output event sequence is finally post-processed into a structured MIDI format using a rule-based event parser, ensuring compliance with MIDI standards. This decoder design assumes that harmonic and stylistic constraints have already been modeled in the latent space during the diffusion process. Therefore, no additional conditional input is introduced at the decoding stage, promoting architectural simplicity and computational efficiency.

### 3.6. Loss Function Design

To enable global optimization and ensure the structural coherence between generated note sequences and harmonic constraints, this study adopts a joint training strategy. Specifically, the VAE, diffusion, and CRF modules are integrated into an end-to-end framework, and their losses are jointly optimized.

The overall loss is defined as:

(21) $$L_{total}=L_{VAE}+\lambda_{1}L_{Diffusion}+\lambda_{2}L_{CRF}$$

where $\lambda_{1}$ and $\lambda_{2}$ are the trade-off coefficients used to balance the contribution of each component.

#### 3.6.1. VAE Loss

To learn structured latent representations from MIDI data, the VAE is trained to maximize the Evidence Lower Bound (ELBO), defined as:

(22) $$L_{VAE}=E_{q\left(z\left|x\right.\right)}\left[logp\left(x\left|z\right.\right)\right]-KL\left(q\left(z\left|x\right.\right)\left\|p\left(z\right)\right.\right)$$

where x denotes the input note sequence, z is its latent embedding, and the loss balances the reconstruction fidelity and latent regularization through the Kullback Leibler (KL) divergence.

#### 3.6.2. Diffusion Model Loss

After obtaining the latent representation from the VAE, a diffusion model is trained to progressively denoise and synthesize structured embeddings. The objective is to minimize the mean squared error (MSE) [28] between the sampled noise ϵ and the predicted noise $\epsilon_{\theta }$, as shown in Equation (23):

(23) $$L_{Diffusion}=E_{\epsilon ,t,z_{t}}\left[{\left\|\epsilon -\epsilon_{\theta }\left(z_{t},t\right)\right\|}^{2}\right]$$

where $z_{t}$ denotes the latent variable at diffusion step t, and $\epsilon_{\theta }$ is the noise predicted by the model.

#### 3.6.3. CRF Loss

The CRF module is trained independently in a pretraining stage, using the pitch-class features extracted from the VAE-encoded sequences. The objective is to maximize the conditional log-likelihood of the correct chord sequence. The partition function *Z*(*y*) is computed via the forward–backward algorithm to ensure proper normalization, and smoothing constants α are applied as described in Section 3.4.1, Section 3.4.2, Section 3.4.3 to handle rare or unseen events. The CRF loss can be formulated as:

(24) $$L_{CRF}=-\left(\sum_{t}\sum_{k}\lambda_{k}f_{k}\left(h_{t},h_{t-1},y_{t}\right)-logΖ\left(y\right)\right)$$

Here, $y_{t}$ denotes the pitch-class feature at time t, $h_{t}$ is the corresponding chord label, $f_{k}$ denotes the predefined entropy-based feature functions, and $\lambda_{k}$ are the learnable weights associated with them, and $Ζ\left(y\right)$ is the partition function ensuring a valid probability distribution. This pretraining ensures that the CRF outputs harmonically informed chord sequences. During the training of the diffusion model, the CRF parameters remain fixed, and the inferred chord embeddings serve as conditioning vectors, providing theory-aware guidance without joint optimization.

## 4. Experiments and Results

### 4.1. Dataset and Parameter Settings

This paper adopts the LMD-matched dataset, a refined subset of the Lakh MIDI Dataset that is aligned with the Million Song Dataset via audio fingerprint matching, resulting in significantly improved alignment quality compared to LMD-full. The dataset contains 45,129 high-quality MIDI files spanning various genres, with pop, rock, and jazz. For harmonic annotation, chord labels were automatically inferred using the music21 toolkit. During preprocessing, MIDI files were segmented into 4/4 bars, discarding bars not equal to four quarter notes. Bars containing more than eight tracks or any track exceeding 64 events were also excluded. Data augmentation involved ±3 semitone modulation and filtering of invalid pitches. A 50-step cosine noise schedule was used in the diffusion process, with training conducted on an RTX 3090 Ti using FP16 mixed precision. A two-stage evaluation framework was employed to assess model performance under both free and conditional generation scenarios.

### 4.2. Chord Confusion Analysis of CRF Strategy

To evaluate the effectiveness of our proposed strategies in chord inference, we design a multi-dimensional confusion matrix heatmap to compare the alignment between predicted chords and ground truth chords across models. Specifically, for each generation round, we generate 10 sequences and concatenate all frame-level chord labels into two long sequences, from which we compute the confusion matrix after filtering zero-only or non-chord rows and columns.

To clarify the confusion matrix in our experiments, each entry $M_{i,j}$ represents the number of frames where the ground truth chord is *i* while the predicted chord is *j*. Diagonal entries indicate correct predictions, whereas non-diagonal entries reflect either misclassifications or musically plausible deviations. For readability, only the most common chord types—major, minor, seventh, quartal, and whole-tone chords—are displayed, highlighting the model’s performance on musically significant chords.

Quartal chords are constructed by stacking perfect fourths (e.g., C–F–B♭), while whole-tone chords consist of notes each a whole tone apart (e.g., C–D–E–F♯–G♯–A♯). These chord types occasionally appear in modern popular and jazz music, and their presence in off-diagonal entries demonstrates the model’s ability to generate non-traditional yet musically plausible harmonies.

Ground truth chords are automatically annotated using the music21 toolkit: each MIDI file is chordified, and bars are analyzed to determine the most likely root and chord type based on the pitches present. Ambiguous bars are smoothed by selecting the most frequent chord label, ensuring consistency in the reference labels.

While the ground truth distribution is biased toward pop, rock, and jazz styles due to dataset selection and music21 inference rules, the entropy-regularized CRF enables the model to retain a small number of non-diagonal but musically reasonable transitions, preserving stylistic diversity. This design allows the model to balance adherence to conventional harmonic rules with creative flexibility, making it suitable for symbolic music generation and AI-assisted composition.

**Figure 3:**
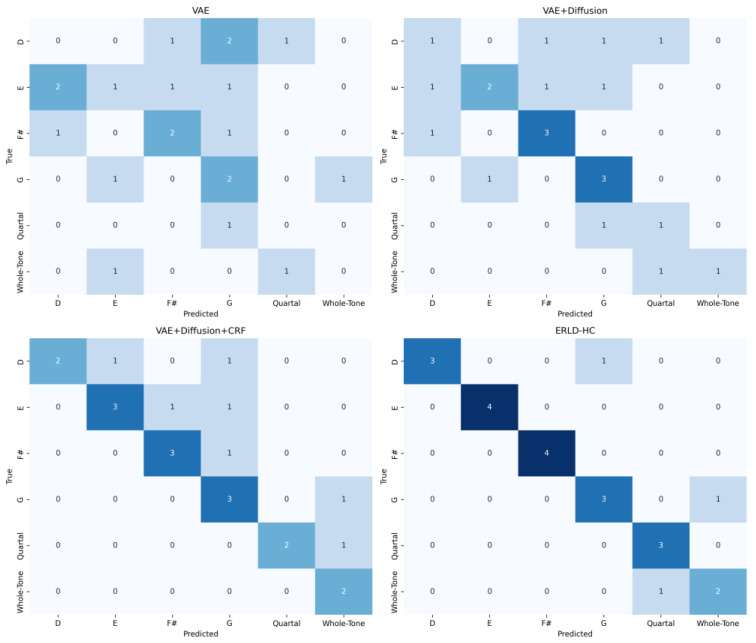
Chord confusion matrix heat map. Darker colors indicate higher frequency of chord prediction matches, while lighter colors indicate lower frequency.

As shown in Figure 3, to prove the validity of the model we proposed, we added modules one by one to conduct ablation experiments. The baseline VAE+Diffusion model exhibits scattered and weak diagonal responses, with a mean diagonal value of 1.83, reflecting poor chord consistency and frequent misclassifications. In contrast, the VAE+Diffusion+CRF model achieves a diagonal mean of 2.5, benefiting from the introduction of global harmonic constraints in the initial stage and temporal dependencies captured by CRF.This significantly reduces illogical harmonic transitions and enhances correct chord alignment. Our model, ERLD-HC, further improves the diagonal consistency to 3.16, and effectively resolves ambiguous transitions such as D → G vs. D → F#, which are commonly confused in baseline models. Notably, ERLD-HC retains a small number of non-diagonal but musically plausible transitions such as G → Whole-Tone, demonstrating a balance between theoretical harmonic correctness and creative diversity. The confusion matrix of ERLD-HC (bottom-right in Figure 3) shows a sparse, sharp diagonal structure, while its few off-diagonal entries reflect stylistic variation rather than noise. Introducing entropy regularization into CRF inference prevents the model from being overly confident in a single chord label, reduces overfitting to deterministic chord transitions, preserves harmonic diversity, and leads to more coherent melodic variations.

In quantitatively assessing alignment, we calculate Kappa coefficient (Cohen’s Kappa) [29] to evaluate the consistency between predicted and actual chord labels and eliminate the influence of random guesses. The calculation formula is Equation (25):

(25) $$k=\frac{p_{o}-p_{e}}{1-p_{e}}$$

where $p_{o}$ represents observation consistency, that is, the sum of diagonal elements is divided by total sample size, and $p_{e}$ represents random consistency, that is, the accuracy rate of expected random guessing, calculated through marginal distribution. The Kappa coefficient is taken between 0 and 1. As shown in Table 1, the 0.83 of our model is better than the 0.61 of VAE+Diffusion+CRF while retaining diversity. Meanwhile, calculate the proportion of chord transitions predicted by the Functional Compliance Rate (FCR) statistical model that conform to the rules of music theory, which is used to quantify the theoretical rationality. The calculation method is the number of compliant transfers divided by the total number of transfers. In this experiment, 86% of ERLD-HC is higher than 68% of VAE+Diffusion+CRF, which is in line with music theory but retains creativity by controlling non-diagonal elements. We calculate the Stylized Retention Rate (SRR) to quantify the proportion of non-theoretical yet artistically valuable chord transitions retained by the model during music generation. Although these transitions do not conform to traditional harmonic rules, they are in line with specific musical styles such as jazz (quartal or whole-tone chords). The SRR is equal to the number of stylized non-diagonal transfers divided by the total number of non-diagonal transfers. The SRR of ERLD-HC is 33%, indicating that the model selectively retains some theoretically inconsistent but reasonable transitions while maintaining a high FCR, which meets the requirements of creative auxiliary tools.

The non-diagonal entries in the confusion matrix may reflect stylistic variations rather than model error. For example, quartal and whole-tone transitions are more prevalent in jazz-influenced MIDI files, while pop and rock files show stricter triadic patterns. Therefore, the proposed CRF features, including entropy-based regularization (PCE, CTE, KME), are designed to be flexible across styles, encouraging harmonic coherence without overly constraining genre-specific variations. Future work could explore style-specific feature adaptation for even finer control.

These results confirm that our entropy-regularized CRF not only improves the accuracy of harmony but also maintains the flexibility of expression in symbolic music generation.

To further evaluate the chord prediction accuracy of each model, we calculate Precision, Recall, and F1-score [30] based on the chord-level confusion matrix constructed from the generated sequences. As shown in Table 2, the baseline VAE+Diffusion model yields an F1-score of 0.51, indicating moderate chord-matching performance. By introducing CRF, the VAE+Diffusion+CRF model achieves a significantly improved F1-score of 0.68. Our proposed ERLD-HC framework achieves the highest scores across all three metrics, with an F1 of 0.82, demonstrating that entropy-regularized chord inference within the diffusion process leads to more precise and consistent harmonic predictions.

### 4.3. Visual Comparison of MIDI Generation Across Models

**Figure 4:**
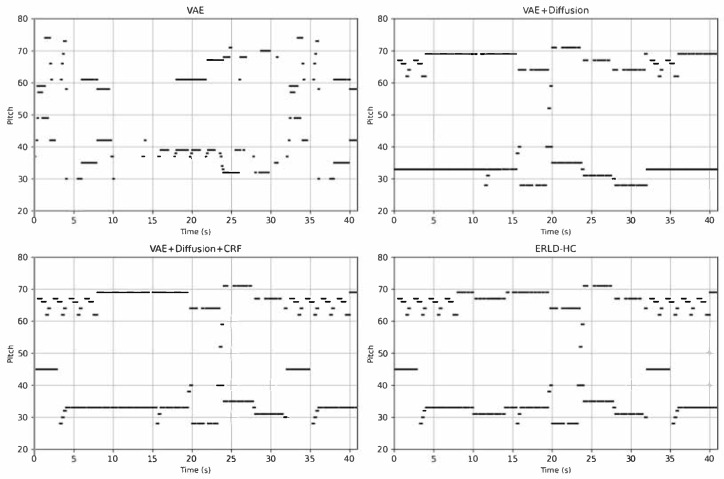
Example piano rolls of experiments. Along the horizontal axis is the time of the generated MIDI, which is 41 s, while pitch values are distributed vertically.

To visualize the MIDI generated by our models, we rendered the piano rolls of two benchmark model, the VAE+Diffusion+CRF model, and our proposed ERLD-HC model, as illustrated in Figure 4. The note sequences generated by the baseline VAE model exhibit a lack of rhythmic structure, abrupt changes, and overall chaotic and incoherent patterns. Although the diffusion-based baseline improves coherence, it suffers from excessive repetition. The VAE+Diffusion+CRF model reduces redundancy but still contains a recurring 10-s repetition. In contrast, our ERLD-HC model displays tighter note groupings, a more balanced pitch distribution, and higher rhythmic entropy—indicating enhanced phrase-level coherence and structural consistency in the generated music.

### 4.4. Design of Evaluation Indicators for Music Generation

In order to verify the Entropy-Regularized Latent Diffusion for Harmony Constrained model designed in this paper, this paper conducts ablation experiments from three dimensions: Harmony, Melody, and Overall Generation Quality, and evaluates the contributions of different modules respectively.

#### 4.4.1. Harmony Assessment

1. Harmony Violation Rate (HVR)

The harmony violation rate (HVR) [31] is an indicator that quantifies the degree of violation of traditional harmony rules (such as part progression, chord connection, dissonant resolution, etc.) in musical works. This indicator analyzes the interval relationship between consecutive chords and counts the frequency of parallel fifths/octaves and interval jumps. The calculation formula can be expressed in Equation (26):

(26) $$HVR=\frac{N_{violation}}{N_{total}}\times 100\%$$

$N_{violation}$ represents the number of violations detected within the analysis range, $N_{total}$ represents the overall analysis of harmony events.

2. Chord Transition Probability (CTP)

Chord transition probability (CTP) [32] describes the transition rules of adjacent chords in MIDI sequences and reflects the statistical characteristics of harmonic progressions. Given chord sequence $\left\{c_{1},c_{2},...,c_{T}\right\}$, its transition probability matrix $Μ\in R^{N\times N}$ is defined in Equation (27):

(27) $${Μ}_{ij}=Ρ\left(c_{t}=j|c_{t-1}=i\right)=\frac{N\left(i\to j\right)}{\sum_{k=1}^{N}N\left(i\to k\right)}$$

Here, N represents the number of chord categories, and $N\left(i\to j\right)$ is the frequency of transitions from chord i to j in the training data. In order to facilitate the comparison of various model indicators, in this paper, chord transition entropy (CTE) H is used instead of chord transition probability. The chord transition frequency is statistically calculated from the LMD dataset, smoothed by Laplace and normalized to avoid zero probability. The chord transition entropy is obtained by calculating the Shannon entropy for the probability distribution P of each row of the transition matrix M and taking the average as shown in Equation (28):

(28) $$H=\frac{1}{N}\sum_{i=1}^{N}\left(-\sum_{j=1}^{N}P\left(c_{j}|c_{i}\right)logP\left(c_{j}|c_{i}\right)\right)$$

3. Chord Saturation (CS)

Chord saturation (CS) quantifies how densely pitches are packed within a chord, helping to capture how full or harmonically tense a chord is perceived to be. High-saturation chords usually contain a large number of notes and have a compact pitch distribution, while low-saturation chords may consist of only a few notes. We define chord saturation as the ratio of the total number of notes in a musical segment to the maximum possible number of notes as shown in Equation (29), where the maximum value is calculated as 12 times the number of chords (assuming that each chord can theoretically cover all 12 pitches). This indicator quantifies the overall harmonic density by measuring the degree to which music utilizes the chromatic scale. Its value close to 1 indicates rich harmonic content, while a value close to 0 indicates less pitch variation:

(29) $$CS=\frac{total\_notes}{max\_possible}$$

#### 4.4.2. Melodic Assessment

1. Pitch Contour Smoothness (PCS)

The smoothness of the pitch contour quantifies how continuously the pitch changes in a melody. A smoother contour implies smaller and more gradual pitch intervals between adjacent notes, which often suggests higher melodic coherence. One proxy for measuring this coherence is the average second-order difference of pitch values. A lower value indicates a more coherent and less jumping melody, while higher values correspond to more abrupt melodic changes. As shown in Equation (30), the pitch sequence $P=\left[p_{1},p_{2},\ldots ,p_{n}\right]$ is extracted from the melody part of a MIDI file, sorted by onset time, and evaluated using a second-order difference within a sliding window.

(30) $$PCS=mean\left(\left|p_{i+2}-2p_{i+1}+p_{i}\right|\right)$$

2. Contour Volatility (CV)

Contour volatility (CV) is an indicator for quantifying the intensity of melodic pitch changes, comprehensively reflecting the amplitude and frequency of directional changes of interval jumps. Unlike smoothness, which focuses on continuity, volatility emphasizes the unpredictability of change and is suitable for evaluating the improvisational nature, decorative complexity or tension of a melody. It is calculated by comparing the number of direction reversals (for example, from rising to falling) with all possible transition numbers as shown in Equation (31). A higher value indicates the presence of complex ornamental notes, while a lower value suggests that the melody moves linearly.

(31) $$CV=\frac{N_{s}}{N-2}$$

N represents the total number of notes, $N_{s}$ represents the number of direction changes, and the denominator N−2 indicates possible combinations of three adjacent notes.

#### 4.4.3. Overall Generation Quality

1. Structural Index (SI)

The structural index (SI) assesses the clarity of musical forms by measuring the degree of separation between clusters in the feature space while considering temporal continuity. Each note in the MIDI sequence corresponds to a sample in the feature matrix $X\in R^{N\times d}$, where N is the number of notes and d is the feature dimension. Features are standardized, then segmented into K clusters using k-means [33]. Since notes in the same musical section tend to have similar features, clusters generally correspond to contiguous time regions, making SI a reflection of temporal structural coherence. The mean silhouette coefficient over all notes is computed as the SI value (Equation (32)):

(32) $$SI=\frac{1}{N}\sum_{i=1}^{N}\frac{b\left(i\right)-a\left(i\right)}{max\left(a\left(i\right),b\left(i\right)\right)}$$

where $a\left(i\right)$ is the average distance from note i to other points in the same cluster, and $b\left(i\right)$ is the average distance from note i to the nearest opposite cluster. The range of SI is between −1 and 1. The higher the value, the clearer the structure.

2. Pitch Naturalness (PN)

Pitch naturalness (PN) measures the smoothness of a pitch sequence. While the concept of pitch naturalness generally encompasses scale conformity and harmonic rules, in this work, we focus on interval statistics as a practical approximation. Given a note sequence $P=\left[p_{1},p_{2},\ldots p_{n}\right]$, calculate the semitone difference ${∆}_{i}=\left|p_{i+1}-p_{i}\right|$ of the note and count the proportion of intervals that satisfy ${∆}_{i}\leq 4$ as shown in Equation (33):

(33) $$PN=\frac{1}{n-1}\sum_{i=1}^{n-1}I\left({∆}_{i}\leq 4\right)$$

I is an indicative function. $PN\in \left[0,1\right]$, higher values indicate greater pitch naturalness.

#### 4.4.4. Baselines for Comparison

To provide a fair evaluation, our experiments include both established and recent models as baselines. Specifically, the baseline VAE corresponds to MusicVAE, a widely used symbolic music model. The VAE+Diffusion baseline follows the architecture of “Symbolic Music Generation with Diffusion Models”, which first introduced diffusion into symbolic music. We further extend this model with a CRF constraint to obtain VAE+Diffusion+CRF, serving as an intermediate variant.

Other recent diffusion-based symbolic music approaches, such as Mustango, Hierarchical Cascaded Diffusion, and MusicLDM, target controllability, long-term structural coherence, or audio-domain generation rather than harmonic rule modeling. Due to differences in datasets, tasks, and evaluation metrics, direct numerical comparison with these models is not feasible.

Additionally, other symbolic music generation frameworks, including Hierarchical Recurrent Neural Networks (HRNN), Structure-Informed Positional Encoding (SIPE), and DeepBach, focus on melody-level structure, long-term coherence, or style-specific generation. Similarly, due to task and dataset differences, we do not provide a quantitative comparison, but a qualitative discussion is provided to contextualize ERLD-HC’s focus.

Qualitatively, Mustango and Hierarchical Cascaded Diffusion emphasize user-driven controllability and structural coherence, producing coherent melodies but without enforcing symbolic-level harmonic rules. MusicLDM, as an audio-domain model, generates natural-sounding music but lacks symbolic harmonic control. In contrast, ERLD-HC focuses on harmonic consistency and stylistic flexibility in symbolic music, producing MIDI sequences that adhere to music-theoretical rules while retaining artistically valuable variations. This qualitative assessment complements the quantitative results presented in Table 3 and Table 4.

#### 4.4.5. Experimental Result

This section quantitatively evaluates the performance of the baseline model, its variants, and the proposed ERLD-HC framework in symbolic music generation, focusing on three key dimensions: harmonic structure, melodic contour, and overall generative quality. To ensure fair comparison, all models generate 50 MIDI sequences of a fixed duration of 41 s, and the reported results are averaged across all generations. Both unconditional and conditional generation settings are assessed. It should be clarified that, in the current framework, harmonic guidance comes solely from the internal CRF inference, rather than any external chord conditioning interface. The diffusion backbone provides a foundation for generative diversity, while the entropy-regularized CRF introduces implicit harmonic rules and temporal dependencies, resulting in outputs that exhibit rich harmonic structure and maintain stylistic consistency. Future work may explore external chord conditioning or guidance-scale mechanisms to provide explicit user-driven harmonic control.

The results, as shown in Table 3 and Table 4, respectively. It should be clarified that Unconditional generation refers to producing music sequences without any input prompts. Conditional generation generates music based on an input MIDI fragment, providing partial harmonic or melodic guidance to the model. During music generation, the input depends on the setting: unconditional generation requires no input, whereas conditional generation takes a MIDI fragment as input. The system currently does not support generation from textual descriptions or titles, but future work could explore such modalities.

In the unconditional generation, the baseline VAE exhibits a relatively high HVR of 4.55% and CV of 0.49, reflecting its limited theoretical constraints in music. Although incorporating a diffusion process (VAE+Diffusion) slightly reduces the violation rate to 3.90%, it leads to an increased CTE of 0.725, indicating that the model gains diversity at the cost of harmonic consistency. The introduction of CRF constraints in VAE+Diffusion reduces the HVR further to 2.65% and lowers the CTE to 0.61, suggesting improved structural coherence.

The ERLD-HC model that we proposed achieves the best performance, with an HVR of only 1.55%, CTE of 0.48, and the highest PN of 0.88 and SI of 0.81, confirming that our hierarchical entropy-regularization constraint can balance creativity and harmony rules.

In conditional generation, that is, in input-based MIDI, ERLD-HC yields the best results, achieving an HVR of 1.30%, CTE of 0.32, and the highest PN of 0.94. These findings underscore the benefit of integrating rule-based modules into probabilistic generative frameworks: the diffusion model provides a foundation for generative diversity, while the CRF with entropy regularization introduces theoretical rules and controls structural fluctuation, resulting in outputs that exhibit rich harmonic structure and maintain stylistic consistency.

Although direct numerical comparison with Mustango, Hierarchical Cascaded Diffusion, and MusicLDM is not feasible due to differing metrics and datasets, qualitatively, these models demonstrate strengths in controllability, multitrack generation, and long-term structure. In contrast, ERLD-HC focuses on harmonic consistency and stylistic flexibility within symbolic music, providing complementary capabilities. Future work will include detailed qualitative and quantitative comparisons on shared benchmarks to better contextualize the performance of ERLD-HC relative to these models.

#### 4.4.6. Subjective Listening Evaluation

To assess the perceptual quality of the generated music, we conducted a small-scale listening test with 20 participants, all with no formal musical training. Each participant was presented with musical pieces generated by four models: VAE, VAE+Diffusion, VAE+Diffusion+CRF, and ERLD-HC. For each participant, the order of the four model-generated fragments was randomized to avoid order effects. Each fragment lasted 16 bars. Listeners rated melodic clarity, harmonic coherence, and overall quality on a 1–5 scale, where 1 indicates poor and 5 indicates excellent.

The average scores are summarized in Table 5. Results indicate that our proposed ERLD-HC model consistently outperforms the baselines in all three perceptual aspects, demonstrating that incorporating entropy-regularized harmonic constraints leads to more coherent and pleasing musical output even for non-expert listeners.

## 5. Conclusions

This paper proposes ERLD-HC, a novel symbolic music generation framework that integrates structural priors with deep generative modeling. Built upon a VAE+Diffusion backbone, the framework introduces an entropy-regularized CRF module into the cross-attention layers of the diffusion model, enabling enhanced control over harmonic progression during generation.

Experimental results show that ERLD-HC consistently improves upon baseline VAE+Diffusion models, particularly in harmonic coherence and melodic smoothness. We also observe that introducing entropy regularization into the CRF inference helps reduce overconfidence in chord labeling, while maintaining the diversity of harmony, and the melodic changes can be more harmonious. This integration allows the model to balance adherence to harmonic rules with stylistic freedom, resulting in outputs that are both structured and expressive.

Furthermore, ERLD-HC demonstrated the validity of combining diffusion models and structured probabilistic models. By embedding CRF inference within the cross-attention mechanism of the diffusion process, the model effectively aligns latent representations with explicit musical structure, resolving both note-level and global harmonic inconsistencies. This design highlights the value of incorporating music-theoretical priors into generative models for future research.

It should be noted that the current form of harmonic control in ERLD-HC is realized through internal CRF-based inference, not through external chord conditioning interfaces. While this does not provide explicit user-driven control, the model achieves implicit harmonic regularization that effectively balances adherence to musical rules with stylistic diversity. The novelty of ERLD-HC lies in embedding entropy-regularized CRF inference into the cross-attention layers of a diffusion-based generative model, which, to our knowledge, has not been explored in symbolic music generation. Future work could integrate external chord conditioning or guidance-scale mechanisms to extend controllability, building upon the implicit harmonic control demonstrated here.

## Figures and Tables

**Table 1:** Analysis of the compliance and artistry of the chord generation model theory based on the confusion matrix.

Model	Kappa	FCR	SRR
VAE	0.076	0.250	0.066
VAE+Diffusion	0.41	0.523	0.000
VAE+Diffusion+CRF	0.61	0.681	0.140
ERLD-HC	0.83	0.863	0.333

**Table 2:** Index analysis of chord confusion matrix.

Model	Precision	Recall	F1
VAE+Diffusion	0.55	0.52	0.51
VAE+Diffusion+CRF	0.76	0.68	0.68
ERLD-HC	0.84	0.82	0.82

**Table 3:** Based on the unconditional generated model indicators in harmony, melody, and overall quality.

Model	Harmony			Melodic		Overall Quality	
	HVR	CTE	CS	PCS	CV	SI	PN
VAE	4.55%	0.648	0.5	8.25	0.49	0.72	0.68
VAE+Diffusion	3.90%	0.725	0.59	8.05	0.45	0.65	0.76
VAE+Diffusion+CRF	2.65%	0.61	0.64	8.30	0.42	0.75	0.74
ERLD-HC	1.55%	0.48	0.68	8.62	0.30	0.81	0.88

**Table 4:** Based on conditionally generated model indicators in harmony, melody, and overall quality.

Model	Harmony			Melodic		Overall Quality	
	HVR	CTE	CS	PCS	CV	SI	PN
VAE	3.13%	0.316	0.56	1.97	0.62	0.66	0.92
VAE+Diffusion	2.70%	0.38	0.6	1.91	0.55	0.6	0.93
VAE+Diffusion+CRF	2.85%	0.35	0.59	2.01	0.57	0.61	0.93
ERLD-HC	1.30%	0.32	0.64	2.16	0.52	0.72	0.94

**Table 5:** Subjective listening evaluation of generated music by non-expert listeners.

Model	Melody (1–5)	Harmony (1–5)	Overall Quality (1–5)
VAE	3.2	2.9	2.9
VAE+Diffusion	3.5	3.2	3.5
VAE+Diffusion+CRF	3.8	3.3	3.6
ERLD-HC	4.1	3.8	4.1

## Data Availability

The data is from the Lakh MIDI dataset. The Lakh MIDI dataset is a collection of 176,581 unique MIDI files, 45,129 of which have been matched and aligned to entries in the Million Song Dataset. Its goal is to facilitate large-scale music information retrieval, based on both symbolic (using the MIDI files alone) and audio content (using information extracted from the MIDI files as annotations for the matched audio files). This paper adopts the LMD-matched dataset. You can obtain it at https://colinraffel.com/projects/lmd/ (accessed on 30 October 2024).
